# Quantification of fetal organ sparing in maternal low-protein dietary models

**DOI:** 10.12688/wellcomeopenres.17124.1

**Published:** 2021-09-02

**Authors:** Patricia Serpente, Ying Zhang, Eva Islimye, Sarah Hart-Johnson, Alex P. Gould

**Affiliations:** 1 Laboratory of Physiology and Metabolism, The Francis Crick Institute, London, NW1 1AT, UK; 2 MRC National Institute for Medical Research, UK, Mill Hill, London, NW7 1AA, UK; 3 Biological Research Facility, The Francis Crick Institute, London, NW1 1AT, UK

**Keywords:** Fetal growth restriction, Developmental origins of health and disease (DOHaD), Small for gestational age (SGA), Intrauterine growth restriction (IUGR), Organ sparing, Brain sparing, Maternal low protein diet, C57BL/6 mice

## Abstract

**Background: **Maternal malnutrition can lead to fetal growth restriction. This is often associated with organ sparing and long-lasting physiological dysfunctions during adulthood, although the underlying mechanisms are not yet well understood.

**Methods: **Low protein (LP) dietary models in C57BL/6J mice were used to investigate the proximal effects of maternal malnutrition on fetal organ weights and organ sparing at embryonic day 18.5 (E18.5).

**Results: ** Maternal 8% LP diet induced strikingly different degrees of fetal growth restriction in different animal facilities, but adjustment of dietary protein content allowed similar fetal body masses to be obtained. A maternal LP diet that restricted fetal body mass by 40% did not decrease fetal brain mass to the same extent, reflecting positive growth sparing of this organ. Under these conditions, fetal pancreas and liver mass decreased by 60-70%, indicative of negative organ sparing. A series of dietary swaps between LP and standard diets showed that the liver is capable of efficient catch-up growth from as late as E14.5 whereas, after E10.5, the pancreas is not.

**Conclusions: **This study highlights that the reproducibility of LP fetal growth restriction studies between laboratories can be improved by careful calibration of maternal dietary protein content. LP diets that induce 30-40% restriction of prenatal growth provide a good model for fetal organ sparing. For the liver, recovery of growth following protein restriction is efficient throughout fetal development but, for the pancreas, transient LP exposures spanning the progenitor expansion phase lead to an irreversible fetal growth deficit.

## Introduction

Moderate nutrient deprivation during animal development results in viable undersized adults. In humans and other mammals, intrauterine growth restriction (IUGR) limits the fetal supply of nutrients and oxygen such that overall growth is decreased but not all organs are equally affected ( Barker & Osmond, 1986; Dobbing & Sands, 1971; Gruenwald, 1963). This non-isometric (asymmetric) scaling down of body parts reflects preferential utilization of scarce nutrient resources by certain tissues such as the brain at the expense of others, such as the liver and pancreas. This process is known as organ sparing and, although it is critical for fetal survival, there is a trade off in terms of the suboptimal functions of both the spared and the non-spared organs later in adult life ( Hales & Barker, 2001; Hanson & Gluckman, 2014; McMillen & Robinson, 2005; Ravelli *et al.*, 1998; Sharma *et al.*, 2016). The mechanisms of organ sparing are not yet well understood but, in the case of the brain, it is known to involve diverting blood flow away from the periphery ( Giussani, 2016; Thornburg, 1991).

Pioneering work in rats by Widdowson and McCance showed that fetal undernutrition has long-lasting effects upon growth trajectories ( Widdowson & McCance, 1963; Widdowson & McCance, 1975). More recently, maternal low-protein diets in rodents have proved useful for investigating how fetal nutrition impacts upon organ growth, function and adult physiology ( Barbeito-Andres *et al.*, 2019; Berends *et al.*, 2018; Bol *et al.*, 2009; Chen *et al.*, 2010; Gould *et al.*, 2018; King *et al.*, 2019; Langley-Evans *et al.*, 1999; Ozanne & Hales, 2004; Tarry-Adkins *et al.*, 2015; Watkins *et al.*, 2008; Zambrano *et al.*, 2006). One study of C57BL/6 mice examined the impact of a low protein maternal diet during pregnancy and/or postnatal stages upon the organ weights of pups at postnatal day 21 (P21) ( Chen *et al.*, 2009). Lowering maternal dietary protein from 20% to 8% during postnatal stages resulted at P21 in significantly smaller kidneys, pancreas, spleen, vastus lateralis, liver and heart but not brain or lungs. In contrast, a low protein (8%) maternal diet during pregnancy, followed by cross-fostering to a standard protein (20%) maternal diet at postnatal stages led to near normal weights at P21 for most organs, although the spleen, heart and thymus were significantly larger ( Chen *et al.*, 2009). This and other studies raise the question of how the protein content of maternal diets during pregnancy affects C57BL/6 organ weights at earlier stages, prior to birth.

Here, we use C57BL/6J mice to investigate the effects of low protein maternal diets upon fetal growth trajectories, organ weights and organ sparing at embryonic day 18.5 (E18.5). Using diet swap experiments, we define critical time windows during pregnancy, when low dietary protein has a long-lasting impact upon the growth of the pancreas and liver. We also document marked differences in fetal growth restriction on low protein diets between different animal houses.

## Methods

### Ethics

Animal studies were performed under a UK Home office approved project license (PAA689E24) and in accordance with institutional welfare guidelines and local ethical committees. All efforts were made to ameliorate any suffering and animals fed a low-protein diet were regularly monitored for health status, and also weighed every other day to confirm there was no excessive loss of body mass. All results are reported in line with ARRIVE 2.0 guidelines ( Percie du Sert *et al.*, 2020).

### Mouse breeding and diets

The C57BL/6J strain of mice was selected as it is inbred and very widely used in genetic studies as well as in models of human diseases. Animals were originally obtained from The Jackson Laboratory and maintained at the MRC National Institute for Medical Research (NIMR), Mill Hill, UK until 2016. From 2016 onwards, C57BL/6J mice were maintained at the Francis Crick Institute (The Crick), London, UK. All mice were housed in the same temperature-controlled room at 21°C with a 12-hour light: dark cycle. Water and food were provided *ad libitum* and natural matings were set up with one male and up to three females, of at least 10 weeks of age and 20g body weight, per cage. Timed pregnancies were performed with the morning of the vaginal plug counted as 0.5d post coitus (E0.5) and then dams were placed using alternate allocation to cages with access to either standard chow (control group) or to a low protein diet (experimental group), with up to a maximum of three per cage. Control and experimental groups of animals are visibly different so cannot be blinded from the experimenter during the conduct of the experiment but subsequent statistical analysis, carried out by different individuals, was blinded. The potential confounding effect of circadian rhythms was minimized by switching diets and harvesting all embryos during a fixed time-window of the day. At NIMR the standard diet was #5021 from LabDiet (21.5% protein) and the low protein diet was #4400 from ABdiets, now #100195 from Altromin (8% protein). At the Crick, the standard diet was #2018S from Envigo laboratories (18.6% protein), the 8% low protein diet (Envigo TD.170638) was custom formulated to be similar to that used at NIMR, and additional isocaloric diets were formulated for 6% protein (Envigo TD.180032), 4% protein (Envigo TD.180031) and 3% protein (Envigo TD.180333). The composition of all diets used in this study is provided in the extended data.

### Embryo and fetal weights

Dams were killed by cervical dislocation at 18.5 days post coitus and death was confirmed by exsanguination. E18.5 embryos were harvested in ice-cold phosphate-buffered saline (PBS), dried on absorbent tissue, and the body weighed with a readability and repeatability of 0.1mg on an XB 120A analytical balance ( Precisa UK). Embryos were photographed in PBS on a Zeiss SV 11 dissecting microscope using a Nikon D700, Lens AF Micro Nikkor 60mm 1:2.8D. Embryos were weighed singly at E10.5, E12.5, E14.5 E16.5 and E18.5 (i.e. the experimental unit is a single animal). However, at E8.5, embryos with 8 somites ( Theiler Stage 13) were selected and weighed from a single litter in groups of 4-8 (i.e. the experimental unit is a litter). For each E18.5 embryo, the brain, liver and pancreas were dissected in ice-cold PBS using watchmaker’s forceps (World Precision Instrument #14096), dried on absorbent tissue and weighed individually on the XB 120A balance. Embryo and organ weights were paired, and the litter of origin recorded. Sample sizes (n≥8) were decided based on previous experimental results and the published literature, except for E8.5 where n≥2 litters. No animals were excluded from the analysis.

### 16S microbiome sequencing

Faecal pellets were collected directly from C57BL/6J mice at six weeks of age maintained at the NIMR or the Crick animal facility on either the #2018S or #5021 diet. Pellets were snap frozen in liquid nitrogen and stored at -80 °C. DNA was extracted using the QIAamp Fast DNA Stool Mini Kit ( Qiagen) and normalised to a concentration of 5μg/μl. V3/V4 specific primers were used to amplify the ~550bp amplicon and DNA was purified using AMPure beads ( Beckman Coulter Life Sciences). A second polymerase chain reaction step was used to attach Illumina index primers ( Illumina) and sequencing adaptors, before purifying the DNA with AMPure beads again. Libraries were measured for purity and quantity on the Nanodrop 1000 ( Thermo Fisher Scientific) before denaturation. Sequencing was carried out on the Illumina Miseq ( Illumina) as per the manufacturer’s instructions for 16s metagenomics sequencing library preparation. The MiSeq provides an on-instrument analysis of the fastq files using the MiSeq Reporter Software, which classifies observed organisms via alignment to the Greengenes database.

### Data analysis and statistical methods

**Figure 1.**
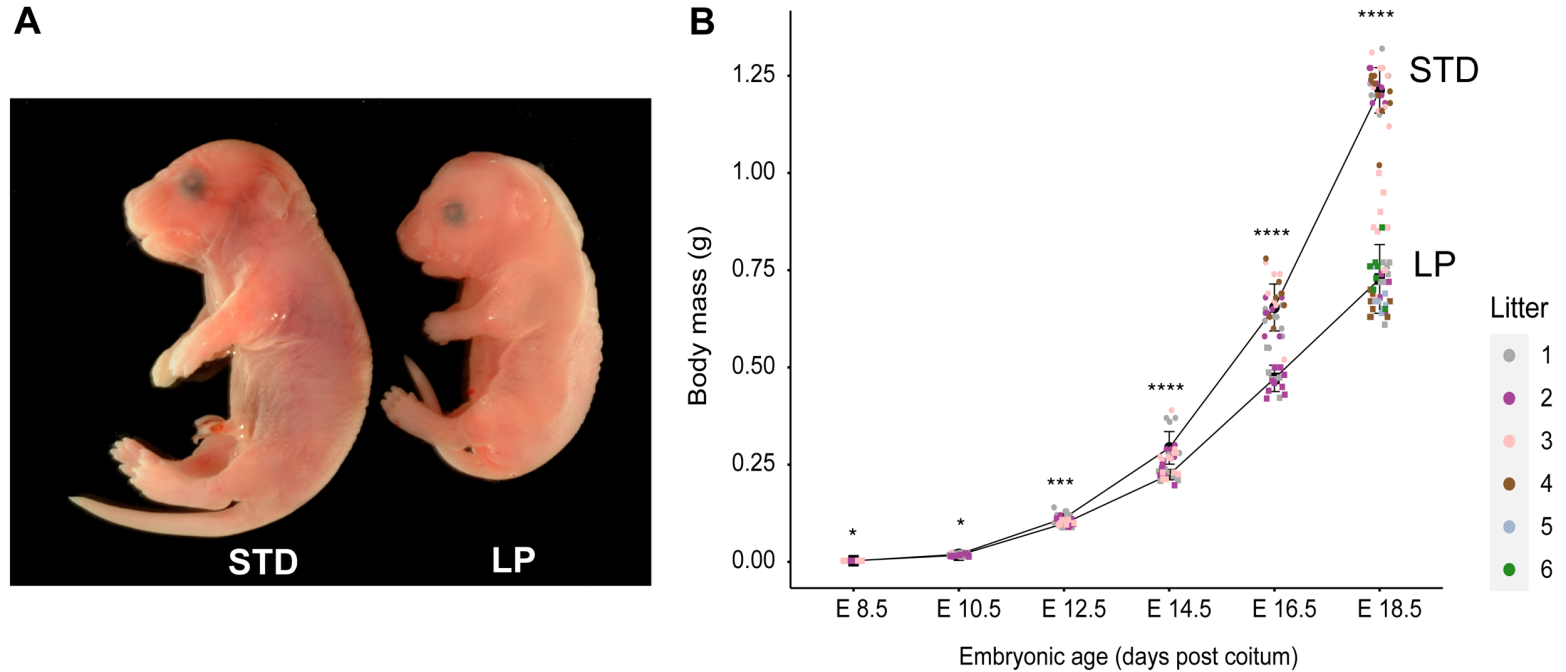
Fetal growth curves for standard (STD) and low protein (LP) maternal diets. ( **A**) The 8% LP maternal diet (#4400) decreased the mass of E18.5 embryos by ~40% relative to the STD diet (#5021). ( **B**) Growth trajectories of embryos from E8.5 to E18.5 on STD and LP diets. In this and all subsequent figures embryos from different litters are indicated with different coloured data points and asterisks indicate statistical significance between STD and LP weights at the indicated stages (* *p* < 0.05, ** *p* < 0.01, *** *p* < 0.001 and **** *p* < 0.0001). C57BL/6J mice were housed in the animal facility at National Institute for Medical Research (NIMR). Details of all diets are provided in Table S1. The source data and statistical analysis for this and all subsequent figures are provided in Table S2 and Table S3 ( Serpente *et al.*, 2021).

All graphs and statistical analyses were generated using RStudio Version 1.2.5042 (2020-04-01). Boxplots were made using the ggplot2 package and show the median with the first and third quartiles of the interquartile range, and whiskers extending from the hinge by 1.5× interquartile range. The plot in Figure 1 was also made using the ggplot2 package, with the black line passing through the mean (large black dot), and the error bars showing the standard deviation (SD). In all graphs, individual data points are coloured according to the independent litter of origin. For statistical analyses, the data were modelled by a linear mixed-effects model (LMM) using restricted maximum likelihood (REML) from the lme4 package, or by a general linear mixed-effects model (GLMM) using maximum likelihood (Laplace Approximation) from the glmm package. Experimental variables such as diet, stages, and sex were allocated as fixed effects, whereas independent litters were allocated as a random effect to take account of within litter and between litter variances. The goodness-of-fit of the model to the data were evaluated using the quantile-quantile (QQ)-plot and QQ-line functions in R. Statistical inference for fixed effects was determined by one-way or two-way analysis of variance (ANOVA), followed by a Wald Chi-Square test using the car package, and adjusted for multiple comparisons using estimated marginal means (EMMs) and corrected with Bonferroni post-hoc tests from the emmeans package. Asterisks on all graphs show statistical significance (* *p* < 0.05, ** *p* < 0.01, *** *p* < 0.001 and **** *p* < 0.0001). The source data used in all graphs are provided in Table S2 ( Serpente *et al.*, 2021). For each graph, the descriptive statistics (mean and SD, or the median and range), statistical approach used, allocation of fixed and random effects, choice of post-hoc ANOVA test and statistical significance are provided in Table S3 ( Serpente *et al.*, 2021).

## Results

### Prenatal growth parameters for the LP maternal diet

**Figure 2.**
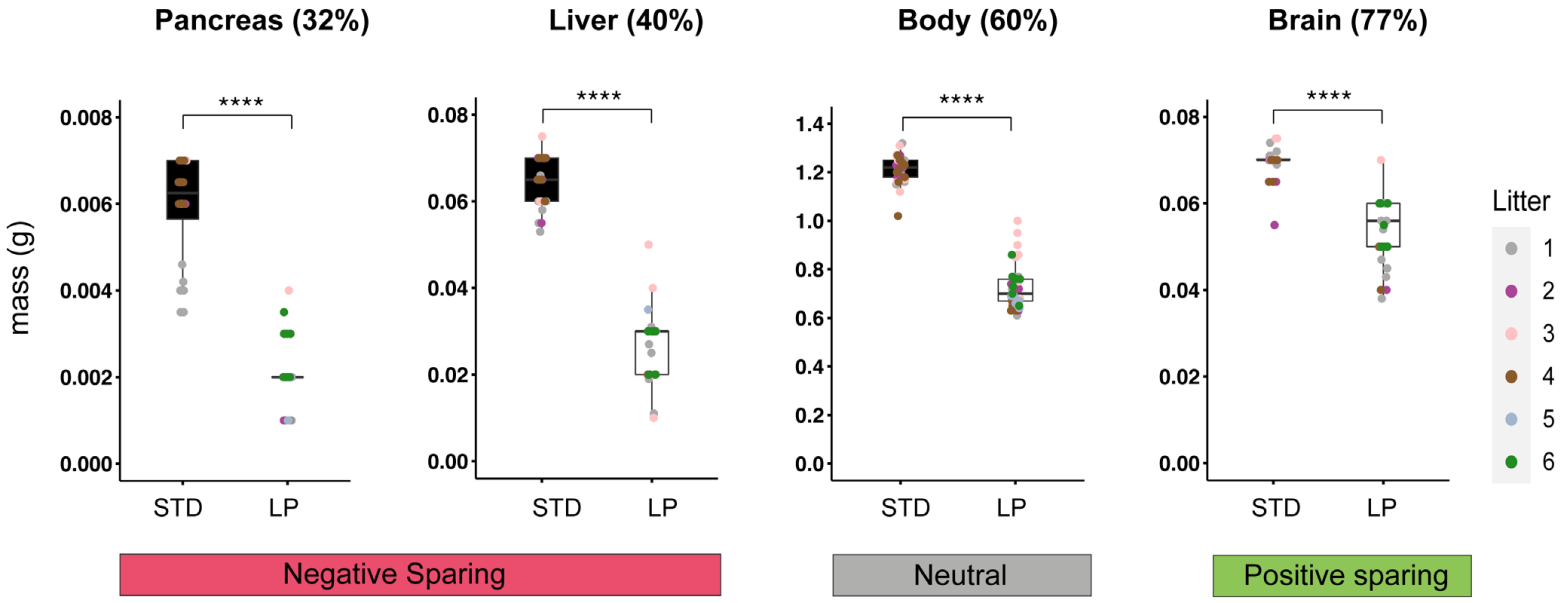
E18.5 body and organ masses for STD and LP maternal diets. Graphs show mass (g) at E18.5 for pancreas, liver, body and brain on STD (#5021) and 8% LP (#4400) maternal diets. For each organ, percentages correspond to LP/STD mass (x100) and positive (green) or negative (red) sparing, relative to the body (neutral, grey), is indicated. Asterisks indicate significant (p<0.0001) differences in weight between STD and 8% LP maternal diets. C57BL/6J mice were housed in the animal facility at National Institute for Medical Research (NIMR). Details of all diets are provided in Table S1 ( Serpente *et al.*, 2021).

C57BL/6J dams were maintained for the duration of pregnancy on either a standard (STD) or low protein (LP) diet, containing 21.5% or 8% protein respectively (Table S1, Serpente *et al.*, 2021). Consistent with previous studies in rats ( Claycombe *et al.,* 2015; Desai *et al.,* 1996), we observed in mice that fetal body size at E18.5 is substantially decreased by LP maternal diet ( Figure 1A). A prenatal time course of body weights revealed that LP maternal diet significantly decreased growth compared to STD diet at all stages from E12.5 to E18.5 ( Figure 1B). At E18.5, this resulted in ~40% lower body weights for LP compared to STD fetuses. We also quantified how LP maternal diet alters organ sizes at E18.5, approximately one day before birth. Accurate masses were determined for the brain, liver and pancreas indicating that all organs from LP fetuses have significantly (p<0.0001) lower masses than their counterparts from STD fetuses ( Figure 2). Non-isometric decreases in organ weights at E18.5 are indicative that prenatal LP induces positive sparing of the brain (77% of STD value) but negative sparing of the liver (40% of STD value) and pancreas (32% of STD value), relative to the body (60% of STD value), which is defined as neutral sparing ( Figure 2). These findings together show that restricting the protein content of the maternal diet throughout pregnancy in C57BL/6 mice results in fetal growth restriction and robust organ sparing.

### Critical windows of maternal protein intake for the growth of the fetal liver and pancreas

**Figure 3.**
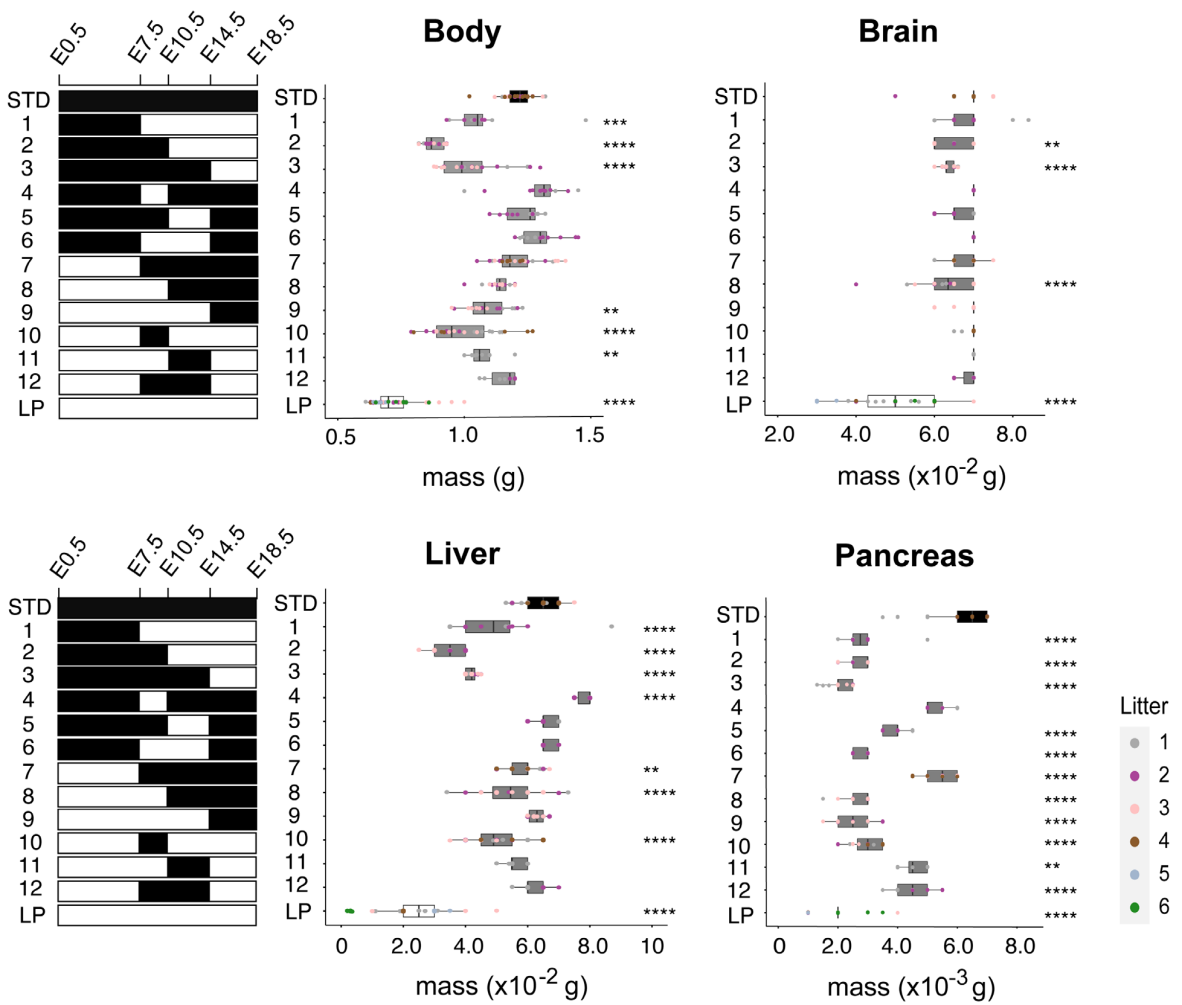
Critical fetal windows for dietary protein-dependent organ growth. Graphs show body and organ (brain, liver, pancreas) masses at E18.5 for maternal STD (#5021) and 8% LP (#4400) diets, or following the indicated series (1-12) of maternal STD-LP diet swaps at E7.5, E10.5 and/or E14.5. Brain mass is decreased substantially by continuous LP but not by shorter LP exposures. In contrast, liver and pancreatic masses are very sensitive to short developmental exposures to LP. Note that the liver but not the pancreas is able to undergo catch up growth to approximately STD size after a late diet swap from LP at E14.5.Asterisks indicate those maternal dietary manipulations with significant differences in body/organ weight from continuous STD diet. C57BL/6J mice were housed in the animal facility at National Institute for Medical Research (NIMR). Details of all diets are provided in Table S1 ( Serpente *et al.*, 2021).

To map the developmental windows during pregnancy when maternal dietary protein intake is the most critical for fetal body and organ masses at E18.5, we utilized a series of twelve dietary interventions ( Figure 3). The maternal diet was switched from STD to LP (interventions 1-3) or from LP to STD (interventions 7-9) at three different fetal stages: E7.5, E10.5 and E14.5. These same fetal stages were also used to delineate transient exposure windows to LP (interventions 4-6) or STD diet (interventions 8-12). For body mass, we observed the expected general tendency to decrease as a function of the duration of fetal exposure to maternal LP diet. A switch from STD to LP diet as late as E14.5 (intervention 3) was sufficient to produce a strong and significant (p<0.0001) decrease in body mass, which is in line with the exponential fetal growth curve ( Figure 1B). Consistent with this, the complementary dietary switch from LP to STD diet at E14.5 (intervention 9) was sufficient to increase body mass significantly (p=0.0012) compared to the continuous LP regime.

Brain mass at E18.5 was largely preserved across all dietary regimes except for continuous LP, where there was a significant (p<0.0001) reduction of ~30%, albeit less than the ~40% decrease of the overall body ( Figure 3). In contrast to brain sparing, liver and pancreas masses at E18.5 were decreased significantly (p<0.0001) following all three switches from STD to LP diet (interventions 1-3, Figure 3). Conversely, after a diet swap from LP to STD at E14.5 (intervention 9), the liver but not the pancreas was able to catch up to approximately normal size ( Figure 3). Strikingly, we also observed that transient windows of LP exposure from either E7.5 or E10.5 until E14.5 (interventions 5 and 6) led to significant (p<0.0001) and substantial decreases in pancreas but not liver mass ( Figure 3). Together, these findings show that fetal growth of the pancreas is more sensitive than that of the liver to short periods of maternal protein deprivation.

### Differential effects of LP diets on fetal growth in different animal facilities

**Figure 4.**
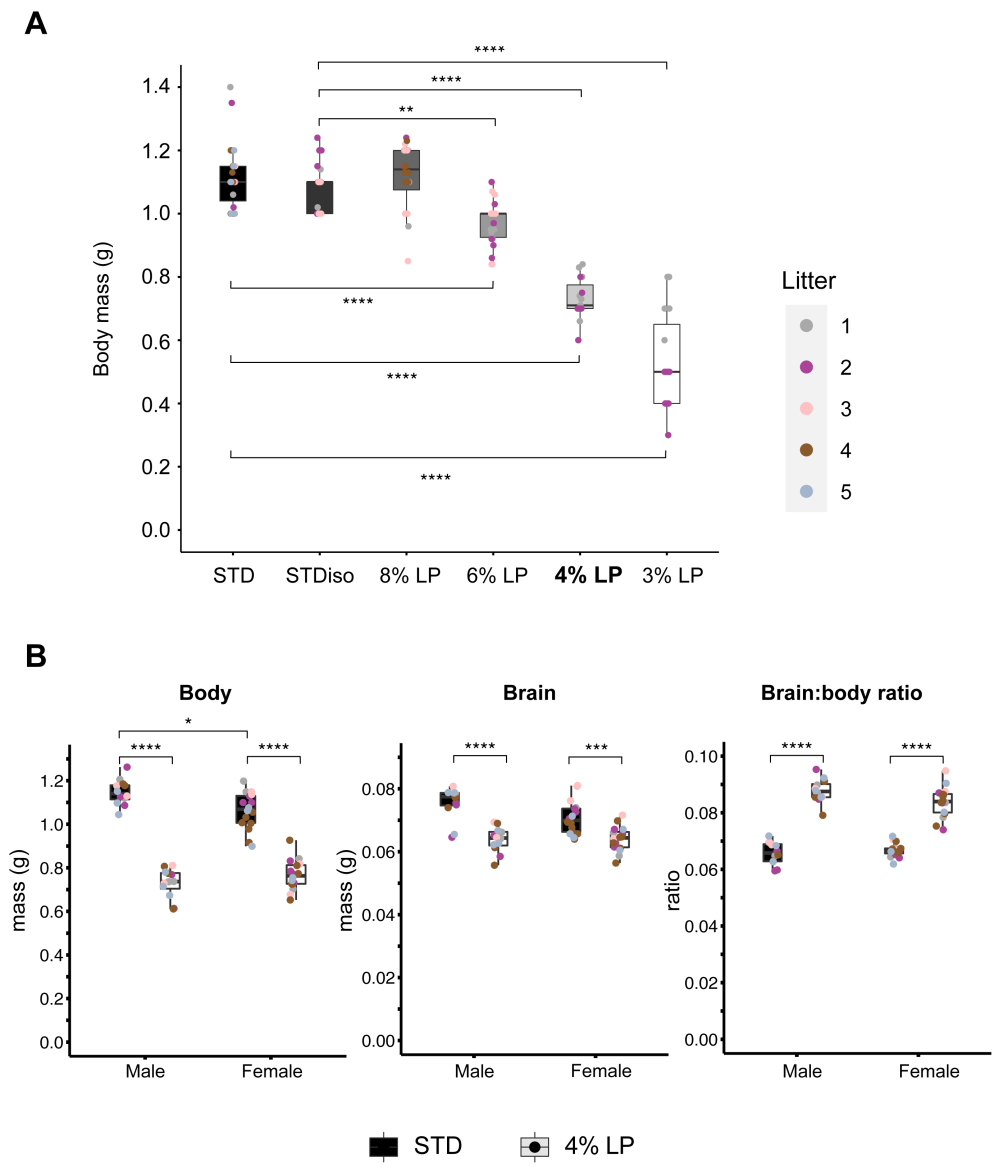
Fetal body masses on standard and low-protein maternal diets. ( **A**) Fetal body weights at E18.5 from dams fed on STD (#2018S) diet or the isocaloric diets STDiso (#TD.180332), 8%LP (#TD.170638), 6%LP (#TD.180032), 4%LP (#TD.180031), or 3%LP (#TD.180033). At the Crick, the 4%LP diet (indicated in bold) approximately recapitulates the percentage decrease in fetal mass observed with the 8%LP diet at NIMR ( Figure 2). ( **B**) Male and female body and brain weights and brain: body ratios on STD and 4% LP maternal diets are comparable at E18.5. Asterisks indicate significant differences in body or organ weight from continuous STD diet. C57BL/6J mice were housed in the BRF at the Crick. Details of all diets are provided in Table S1.

During the course of this study, we moved animal facilities from the former MRC National Institute for Medical Research to the Francis Crick Institute. At the Crick, we used the same strain of mice (C57BL/6J) and an identical LP diet with 8% protein but we were unable to replicate the ~40% fetal growth restriction observed at NIMR. Surprisingly, at the Crick, the 8% LP diet had no significant effect upon E18.5 body mass ( Figure 4A). It is not clear which factor(s) are responsible for the observed difference in the fetal growth response to protein restriction between NIMR and the Crick. However, 16S sequencing of the fecal microbiome of the C57BL/6J colony did reveal a substantial difference in the composition of the major bacterial phyla at the Crick compared to NIMR. In particular, although swapping STD diets (#5021 and #2018S, Table S1, Serpente *et al.*, 2021) at NIMR did not substantially change the adult C56Bl/6J microbiome, switching between the NIMR and Crick animal facilities led to a striking decrease in Bacteriodetes with a concomitant increase in Firmicutes and Tenericutes (Figure S1, Serpente *et al.*, 2021). It is therefore possible that a change in the fecal microbiome contributed to the different severities of maternal protein restriction in the two animal facilities.

To replicate, at the Crick, the ~40% fetal growth restriction observed at NIMR, we compared a STD diet with five isocaloric diets formulated to contain 18.6%, 8%, 6%, 4% or 3% protein (Table S1, Serpente *et al.*, 2021). The STD and STDiso diets (#2018S and #TD.180332) both gave comparable body masses of ~1.1g at E18.5, indicating that the small variation in energy content (3.1 versus 3.5 Kcal/g) between these diets has a negligible effect on fetal growth ( Figure 4A). For body mass at E18.5, the 6% LP diet gave a mild 12.5% decrease, but the 3% LP diet gave a very strong decrease of 52% ( Figure 4A). With the latter diet, however, the spread (variance) of pup weights was greater than that seen at higher protein contents. Hence, the 4% LP diet at the Crick was optimal in terms of achieving a significant (p<0.0001) and substantial (30-40%) decrease in fetal body mass that was close to the ~40% observed at NIMR with the 8% LP diet, yet without the increased variance observed with the 3% LP diet ( Figure 4A–B). Consistent with this, the 4% LP diet gave a pronounced increase in the body: brain ratio indicative of brain sparing, and this was similar between males and females ( Figure 4B). We conclude that replicating the extent of fetal growth restriction between different animal facilities requires careful calibration of the protein content of maternal LP diets.

## Discussion

### Organ-specific temporal requirements for dietary protein

This fetal study shows that LP maternal diet in mice can induce substantial growth restriction and organ sparing prior to birth. Comparing our results with those of a previous LP study of C57BL/6 postnatal weights ( Chen *et al.*, 2009), suggests that the fetal growth restriction we measured at E18.5 could be rescued as early as P21 by postnatal catch-up growth on a STD diet. Within the fetal developmental window itself, catch-up growth has not been well studied for the body or for individual organs but our panel of maternal dietary swaps provide new insights. Fetal body mass can catch up fully by E18.5 with an LP to STD switch at E7.5 but progressively later switches incur increasing weight deficits. For the brain, we found that mass was spared, relative to the body, across all dietary regimes. The 20-30% decrease in brain mass observed with the continuous LP regime is broadly consistent with earlier studies showing that dams fed an 8% protein diet for 1 or 2 months prior to conception and throughout gestation gave birth to young with lower cerebral weights but, interestingly these had similar amounts of cerebral DNA ( Nehrich & Stewart, 1978). This suggests that, overall, cell number in the fetal brain may be more spared from maternal protein restriction than cell volume. At postnatal stages, however, undernutrition is known to decrease the numbers of glial cells ( Leuba & Rabinowicz, 1979).

In contrast to the brain, we found that pancreas and liver masses are sensitive to even short fetal exposures to LP. For the liver, LP to STD diet switches even as late as E14.5 highlighted an impressive capacity for catch-up growth. For the pancreas, however, these experiments revealed that the ability to catch up growth after protein restriction is lost at some point between E7.5 and E10.5. In this regard, it is interesting that pancreas but not liver size is known to be fixed early in fetal development and constrained by the size of the progenitor cell pool ( Stanger *et al.*, 2007). More specifically, all of the Pdx1-expressing progenitors required to make the pancreas are generated from E8.5 to E12.5 ( Stanger *et al.*, 2007). We therefore surmise that maternal LP experienced by the fetus during the window of progenitor expansion may limit the size of the progenitor pool, in turn leading to a long-lasting deficit in pancreas size. This may be one contributing factor explaining the observed association between poor fetal growth and adult pancreatic malfunction ( Hales & Barker, 2001).

### Calibration of dietary protein is important for standardization of fetal growth restriction

A striking finding of our study was that the 8% LP diet decreased the fetal body mass of C57BL/6J mice by 40% at NIMR, yet it had little or no effect at the Crick. The protein content of the diet had to be titrated down to 4% in order to get a comparable amount of fetal growth restriction at the Crick. It remains unclear which differences between animal facilities are relevant but substantial changes in microbiota were observed and could be a contributing factor. Regardless of the underlying cause, our findings highlight that variability between animal facilities poses a serious problem to the reproducibility of maternal nutrition studies in rodent models. One step towards improving reproducibility is to titrate the protein content of isocaloric diets to a level that replicates a standard decrease in fetal body mass, such as the 40% that was used in this study. In conclusion, our LP study highlights one aspect of the much wider challenge of environmental standardization in animal experiments ( Richter *et al.*, 2009).

## Data availability

### Underlying data

Figshare: Extended data for "Quantification of fetal organ sparing in maternal low-protein dietary models". https://doi.org/10779/crick.c.5532651.v2

This collection contains the following underlying data:

   -   Table S1 csv files
   -   Table S2 csv files
   -   Table S3 csv files

### Extended data

Figshare: Extended data for "Quantification of fetal organ sparing in maternal low-protein dietary models". https://doi.org/10779/crick.c.5532651.v2

This collection contains the following extended data:

   -   FigS1.ai – Figure S1
   -   Serpente Table S1
   -   Serpente Table S2
   -   Serpente Table S3

Data are available under the terms of the Creative Commons Attribution 4.0 International license (CC-BY 4.0).

### Reporting guidelines

ARRIVE Compliance Questionnaire and ARRIVE Guidelines 2.0: author checklist are both deposited at Figshare: Extended data for "Quantification of fetal organ sparing in maternal low-protein dietary models". https://doi.org/10779/crick.c.5532651.v2
