# Supplementary material for: Quantification of fetal organ sparing in maternal low-protein dietary models
Source: Wellcome Open Res. 2022 May 4;6:218. Originally published 2021 Sep 2. [Version 2] doi: 10.12688/wellcomeopenres.17124.2 (PMC9120932; doi:10.12688/wellcomeopenres.17124.2)
Supplement: Peer review history

# Peer review: Reviewer response for version 2

**DOI:** 10.21956/wellcomeopenres.19785.r50291

**Published:** 2022-05-19

**Authors:** David J. Hill

**Affiliations:** 1 St Joseph Health Care, Lawson Health Research Institute, ON, Canada

The revision of May 4th 2022 has greatly improved the paper. There are a number of interesting experimental leads that can now be followed up by the authors or others.

Is the work clearly and accurately presented and does it cite the current literature?

Partly

If applicable, is the statistical analysis and its interpretation appropriate?

Yes

Are all the source data underlying the results available to ensure full reproducibility?

Yes

Is the study design appropriate and is the work technically sound?

Yes

Are the conclusions drawn adequately supported by the results?

Yes

Are sufficient details of methods and analysis provided to allow replication by others?

Yes

Reviewer Expertise:

NA

I confirm that I have read this submission and believe that I have an appropriate level of expertise to confirm that it is of an acceptable scientific standard.

# Peer review: Reviewer response for version 1

**DOI:** 10.21956/wellcomeopenres.18915.r48186

**Published:** 2022-02-07

**Authors:** Paula N. Gonzalez

**Affiliations:** 1 Faculty of Natural Sciences and Museum, Anthropology Division, CONICET, National University of La Plata, La Plata, Argentina; 2 Unidad Ejecutora de Estudios en Neurociencias y Sistemas Complejos (ENYS). CONICET, Universidad Nacional Arturo Jauretche, Hospital el Cruce Dr. Nestor Kirchner, Florencio Varela, Argentina

The study by Serpente and co-authors showed a differential effect of maternal low protein diet during gestation on body and organ weight in a mouse model, with the brain positive spared and the liver and the pancreas more affected. The study also explored the critical windows for each organ during prenatal growth, confirming that the timing and extension of nutrient restriction induce different responses across organs.

 In particular, the results suggest that the pancreas is more sensitive than the liver to short periods of maternal protein deprivation. Although the mechanisms of organ sparing were not examined, the authors suggest that the timing of cellular processes such as the early proliferation of progenitors in the pancreas could partly explain the narrower window for a catch-up of this organ compared to the liver. The effect of the redistribution pattern of fetal circulation in IUGR also need to be discussed (e.g., Tchirikov et al., 2002; Ebbing et al., 2009) ^1^^,^^2^

 Interestingly, the effect of an 8% protein diet differs between two animal facilities, which is associated with differences in the microbiome. A review of the literature on experimental models of nutrient restriction reveals variable and even some contradicting results, and this study provides one possible explanation for such differences that deserves further research. This finding is relevant not only for replicating controlled conditions in experimental designs but also for the implications for studying the effect of malnutrition in human populations.

 A previous study with a similar design shows that the effect of low protein diets is not homogeneous across cell types or brain regions (Barbeito *et al*., 2019). Given the detailed analysis of critical windows performed in this study, it would be interesting to explore whether they induce different responses at the cell level beyond the effect on overall brain size.

Is the work clearly and accurately presented and does it cite the current literature?

Partly

If applicable, is the statistical analysis and its interpretation appropriate?

Yes

Are all the source data underlying the results available to ensure full reproducibility?

Yes

Is the study design appropriate and is the work technically sound?

Yes

Are the conclusions drawn adequately supported by the results?

Yes

Are sufficient details of methods and analysis provided to allow replication by others?

Yes

Reviewer Expertise:

Have experience studying the effects of maternal malnutrition on fetal growth, both in human populations and mice.

I confirm that I have read this submission and believe that I have an appropriate level of expertise to confirm that it is of an acceptable scientific standard.

## References

[1] : Redistribution pattern of fetal liver circulation in intrauterine growth restriction. *Acta Obstet Gynecol Scand* .2009;88(10) : 10.1080/00016340903214924 1118-23 10.1080/00016340903214924 19707895

[2] : Liver blood perfusion as a possible instrument for fetal growth regulation. *Placenta* .2002;23 Suppl A: 10.1053/plac.2002.0810 S153-8 10.1053/plac.2002.0810 11978076

# Response

**Published:** 2022-04-22

**Authors:** Alex Gould

**Affiliations:** The Francis Crick Institute, UK

*The study by Serpente and co-authors showed a differential effect of maternal low protein diet during gestation on body and organ weight in a mouse model, with the brain positive spared and the liver and the pancreas more affected. The study also explored the critical windows for each organ during prenatal growth, confirming that the timing and extension of nutrient restriction induce different responses across organs.*

*In particular, the results suggest that the pancreas is more sensitive than the liver to short periods of maternal protein deprivation. Although the mechanisms of organ sparing were not examined, the authors suggest that the timing of cellular processes such as the early proliferation of progenitors in the pancreas could partly explain the narrower window for a catch-up of this organ compared to the liver.*

*The effect of the redistribution pattern of fetal circulation in IUGR also need to be discussed (e.g., Tchirikov et al., 2002; Ebbing et al., 2009)*

 We thank the Reviewer for these useful comments and have now expanded the first paragraph of the Introduction to discuss the redistribution pattern of fetal circulation. This now includes the evidence from Tchirikov *et al*., 2002 and Ebbing *et al*., 2009 that hepatic blood flow during IUGR may be a causal contributing factor to the restricted growth of multiple organs. In response to both Reviewers comments, we have also cited many other new references to the Introduction and Discussion sections of our rather short initial manuscript in order to better represent the literature.

*Interestingly, the effect of an 8% protein diet differs between two animal facilities, which is associated with differences in the microbiome. A review of the literature on experimental models of nutrient restriction reveals variable and even some contradicting results, and this study provides one possible explanation for such differences that deserves further research. This finding is relevant not only for replicating controlled conditions in experimental designs but also for the implications for studying the effect of malnutrition in human populations.*

 We thank the Reviewer for acknowledging that our finding of a very marked difference in the extent of  IUGR between two animal facilities is interesting and deserves further research. Although it is beyond the scope of the present manuscript, identifying the underlying microbiota or other mechanistic basis for this will be important and, as the Reviewer says, may also have implications for human IUGR. 

*A previous study with a similar design shows that the effect of low protein diets is not homogeneous across cell types or brain regions (Barbeito et al., 2019). Given the detailed analysis of critical windows performed in this study, it would be interesting to explore whether they induce different responses at the cell level beyond the effect on overall brain size.*

 This is a very interesting point. The regional bias in brain sparing shown in the study that we had previously cited by Barbeito-Andres *et al.* 2019 is important and we have now expanded upon this specific point in the Discussion. This includes mentioning the finding by Barbeito-Andres *et al*. 2019 that the volume of the cerebral cortex is more highly spared from the effects of maternal protein restriction than that of the cerebellum. Although it is beyond the scope of our current paper, we will in future investigate in cellular and molecular detail how critical temporal windows of LP exposure impact embryonic neurogenesis in different regions of the brain.

# Peer review: Reviewer response for version 1

**DOI:** 10.21956/wellcomeopenres.18915.r46693

**Published:** 2021-11-02

**Authors:** David J. Hill

**Affiliations:** 1 St Joseph Health Care, Lawson Health Research Institute, ON, Canada

The study showed that maternal 8% LP diet induced substantial growth retardation in fetal mice starting at E12.5 with the pancreas and liver being most affected and the brain relatively spared. However, in a second animal facility, it required a 4% LP diet to get equivalent organ and body weight changes. The microbiome of the mothers differed between the two facilities. A series of dietary transitions between LP and standard diets throughout gestation showed that the liver was capable of catch-up growth from as late as E14.5 but after E10.5, the pancreas is was destined to be growth retarded.

 This paper confirms the impact of maternal LP diet on fetal and organ size that has been reported many times in both mouse and rat. It adds some new data on the ontogeny of the retardation of fetal weight in utero and the critical windows for organ impact between pancreas and liver. The paper also shows that the impact of LP diet on fetal development is not just dependent on animal strain but can be affected by animal facility, and that the protein content of the LP diet may need to be titrated between different institutions to get equivalent effects on organ phenotype. This may or may not be related to maternal microbiome. The paper, therefore, adds new data but is somewhat limited on mechanism.

 The various LP diets used were isocalorific but was the methionine content normalized?

 While the pancreatic weight was especially sensitive to LP exposure, was there an equivalent decrease in pancreatic endocrine cell mass as measured by islet mass? Within the islets was the make-up of all endocrine cells equally affected at E18.5, or was the beta cell contribution particularly vulnerable? This might indicate key ontogeny points at which the lineage development of the beta cells are substantially compromised.

 While brain weight is spared in the embryos after LP exposure, what about neuronal development? There may be long-term neurological deficits which could be supported or refuted in the Discussion from previous literature.

 As the microbiome was measured the Discussion could include any evidence that this is directly connected via maternal physiology to fetal organ development.

Is the work clearly and accurately presented and does it cite the current literature?

Partly

If applicable, is the statistical analysis and its interpretation appropriate?

Yes

Are all the source data underlying the results available to ensure full reproducibility?

Yes

Is the study design appropriate and is the work technically sound?

Yes

Are the conclusions drawn adequately supported by the results?

Yes

Are sufficient details of methods and analysis provided to allow replication by others?

Yes

Reviewer Expertise:

Have experience with low protein diet administered to pregnant mice and rats and the development of the endocrine pancrease in the offspring before and after birth.

I confirm that I have read this submission and believe that I have an appropriate level of expertise to confirm that it is of an acceptable scientific standard, however I have significant reservations, as outlined above.

# Response

**Published:** 2022-04-22

**Authors:** Alex Gould

**Affiliations:** The Francis Crick Institute, UK

*The study showed that maternal 8% LP diet induced substantial growth retardation in fetal mice starting at E12.5 with the pancreas and liver being most affected and the brain relatively spared. However, in a second animal facility, it required a 4% LP diet to get equivalent organ and body weight changes. The microbiome of the mothers differed between the two facilities. A series of dietary transitions between LP and standard diets throughout gestation showed that the liver was capable of catch-up growth from as late as E14.5 but after E10.5, the pancreas is was destined to be growth retarded.*

*This paper confirms the impact of maternal LP diet on fetal and organ size that has been reported many times in both mouse and rat. It adds some new data on the ontogeny of the retardation of fetal weight in utero and the critical windows for organ impact between pancreas and liver. The paper also shows that the impact of LP diet on fetal development is not just dependent on animal strain but can be affected by animal facility, and that the protein content of the LP diet may need to be titrated between different institutions to get equivalent effects on organ phenotype. This may or may not be related to maternal microbiome. The paper, therefore, adds new data but is somewhat limited on mechanism.*

 We thank the Reviewer for their accurate summary and for acknowledging that this paper adds new data. We do also agree that our paper is somewhat limited on mechanism but we wanted to alert the research community at the earliest possible point to our observation that identical mouse genotypes and diets could produce radically different fetal growth responses in different animals houses – and also to offer a practical solution (titration of dietary protein) to minimize such differences and thus to help standardize the degree of fetal growth restriction.

*The various LP diets used were isocalorific but was the methionine content normalized?*

 The Reviewer raises an interesting point. Dietary methionine restriction is known to produce metabolic responses, such as protection against high-fat diet obesity, that are different from those of diets restricted in other essential amino acids. In our study, the methionine content was not equalised between LP isocalorific diets but was proportional to protein content  (Table S1). We therefore do not currently know how many of the LP effects are mediated by de facto restriction of methionine or indeed any of the other individual amino acids. Our diets used dietary casein as the protein source and this was supplemented by a small amount of methionine, in proportion to the protein content of the diet (Table S1). Thus, for the “optimised” 4% LP diet, there is a total methionine content of ~0.21%. This value is thought to lie within the ideal range for dietary methionine restriction (0.17%-0.25%) and is not, in itself, sufficient to evoke essential amino acid deprivation responses, at least in adult male C57BL6/J mice (L. Forney et al. 2017. PMID: 28261952; H. Fang et al. 2021. PMID: 34444768). Future mechanistic studies using LP diets supplemented with sulphur amino acids will reveal the extent to which methionine restriction accounts for our observed patterns of organ sparing. We have now added this interesting point in the first paragraph of the Discussion, citing both references above. In response to both Reviewers comments, we have also cited many other new references to the Introduction and Discussion sections of our rather short initial manuscript in order to better represent the literature.

*While the pancreatic weight was especially sensitive to LP exposure, was there an equivalent decrease in pancreatic endocrine cell mass as measured by islet mass? Within the islets was the make-up of all endocrine cells equally affected at E18.5, or was the beta cell contribution particularly vulnerable? This might indicate key ontogeny points at which the lineage development of the beta cells are substantially compromised.*

 The LP sensitivity of the pancreas provides a striking contrast to the tissue that is the main focus of our laboratory - the brain. We are now concentrating on identifying which cell types are spared in the developing brain and regret that we do not have sufficient resources to also study the pancreas - which is also fascinating.

 The Reviewer raises a very interesting point about whether different cell types in the endocrine and exocrine pancreas are equally spared or not at E18.5. This will be very exciting for another lab to follow up and we have now discussed this point in the Discussion (third paragraph), as well as speculating that the early critical time-window of LP exposure for the pancreas suggests that the expansion of  Pdx1 ^+^ common progenitors of endocrine and exocrine pancreas may be affected.

 More generally, we acknowledge that our initial manuscript was rather short and did not do justice to the literature on the effects of maternal LP models in rodents upon pancreas development and function. We have therefore expanded the Discussion (third paragraph) to cite some of these key findings with 6 new references in this area.

*While brain weight is spared in the embryos after LP exposure, what about neuronal development? There may be long-term neurological deficits which could be supported or refuted in the Discussion from previous literature.*

 The Reviewer raises a fascinating question that we do intend to explore in detail at the cellular and behavioural level in future studies (also see our response to the point above). There is already good evidence that LP brain sparing in rodents is imperfect at the functional level-resulting in subtle to major cognitive and neurodevelopmental abnormalities. At the cellular level, deficits in hippocampal neurogenesis and neuronal number have been observed in various rodent models of IUGR. We did not discuss the literature on this in sufficient detail in our rather concise original Introduction and this has now been remedied in the first paragraph of the revised Introduction, where 5 references are cited for this point (4 of which are new additions).

*As the microbiome was measured the Discussion could include any evidence that this is directly connected via maternal physiology to fetal organ development.*

 We thank the reviewer for this very sensible suggestion and have now added a section on this and three new references to the last paragraph of the Discussion. This includes citing references in support of the idea that the maternal microbiota provides metabolites and substrates essential for fetal growth and these that are transmitted transplacentally to the fetus. We also speculate that a maternal LP diet could influence the representation of Bacteroidetes in the microbiome.
